# Aphalangia possibly linked to unintended use of finasteride during early pregnancy

**DOI:** 10.4103/0256-4947.51805

**Published:** 2009

**Authors:** Bahauddin I. Sallout, Khalid A. Al Wadi

## To the Editor:

Finasteride (Proscar) is a 5-alpha reductase inhibitor used for treatment of benign prostatic hyperplasia.^1^ Finasteride is not FDA-approved for use in women,^2^ but in practice is prescribed with warnings about becoming pregnant. A teratogenic effect on the human fetus has not been reported, but animal studies show external genital abnormalities in male fetuses exposed to the 5-alpha reductase inhibitor in utero so the drug is classified in FDA pregnancy category X (studies in animals or pregnant women have demonstrated positive evidence of fetal abnormalities) and is contraindicated in women who are or may become pregnant.^3^,^4^ We report a case of unintended use of finasteride during early pregnancy. The pregnancy ended with the normal delivery of a baby girl with unilateral hand and toe deformities. A 41-year-old woman, gravida7 para6, was urgently referred to the Maternal-Fetal Medicine (MFM) clinic at King Fahad Medical City. She was seeing a dermatologist due to male pattern alopecia after her last delivery. Finasteride 1 mg was prescribed, and she was warned against pregnancy. However, she did not use contraception because she was breastfeeding and thought she would not become pregnant. Unfortunately, pregnancy was confirmed after a history of 6 weeks of amenorrhea. Finasteride was stopped immediately. She had six previous healthy children and was not known to have any chronic medical illnesses. The family history was unremarkable for congenital anomalies or genetic diseases. She used no medication other than finasteride and there was no history of radiation exposure.

**Figure 1:**
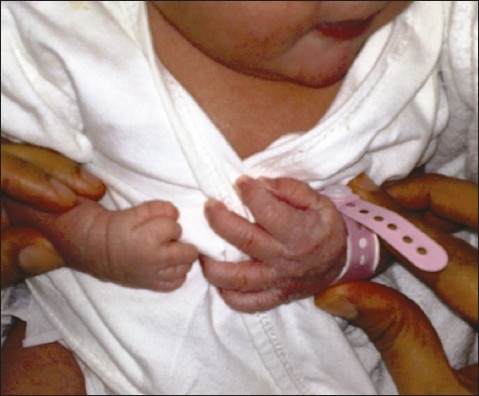
Note the small right hand with short fingers.

**Figure 2:**
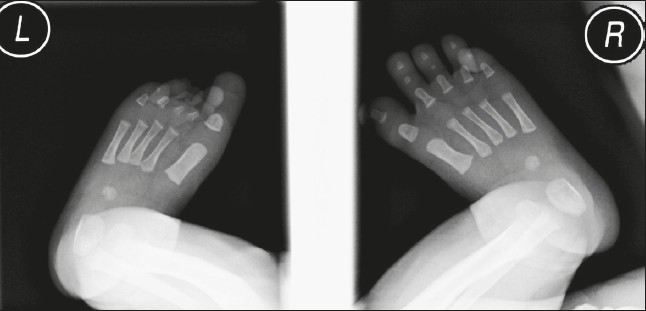
X-ray of both feet. Note the short toes on the left foot with absent phalanges in the second and third toes.

She was counseled about its general and specific (abnormalities of external male genitalia) teratogenic effects of finasteride during the current pregnancy. Ultrasound examination was performed at 12 weeks of gestation (for nuchal translucency, nasal bone and ductus venosus), and was unremarkable. A detailed anatomy ultrasound scan at 18 and 26 weeks of gestation found no anomalies. Because the fetus was a female, the mother was reassured. For the rest of her antenatal care, the pregnancy was uneventful. At term, she delivered a baby girl with a normal birth weight of 3850 g (appropriate for gestational age), a height of 53 cm (at 50th percentile), a head circumference of 34 cm (above 10th percentile), a chest circumference of 33 cm, and abdominal girth of 33 cm. The baby was found to have deformities in the right hand in the form of a small hand with short fingers and absent phalangial bones in all five fingers (aphalangia) (Figure 1), and in the left foot in the form of short second and third toes with absent distal phalanges. Two cafe au lait spots were seen also on the back. No other abnormalities could be identified. X-ray examinations of upper and lower limbs confirmed the clinical findings. Abdominal ultrasound examination was unremarkable (Figure 2).

Limb formation begins at the fourth week of embryonic life. The limb primordium bulges from the body wall consisting from mesoderm covered by a layer of ectoderm.

An apical ectodermal ridge (AER) is a thickened ectoderm located at the apex of the limb. Animal studies have confirmed that removal of the AER results in the arrest of limb development. The degree of deformity depends on the time of insult; the more mature the limb bud, the more the skeletal element forms after AER removal.^5^ In the current case, x-ray of both hands showed absent phalangial bones in the right with normal phalanges in the left, and normal metacarpal bones in both. The carpal bones are normally not noticeable in the x-ray at this age. It is known that ossification of the phalanges takes place antenatally, while the carpal bones ossify postnatally.^6^ Limb anomalies can usually be diagnosed by antenatal ultrasound scan; nevertheless, we missed the diagnosis antenatally, mainly due to lack of suspicion for such potential deformities with the use of finasteride in pregnancy. The appearance of one normal hand could be misleading and result in missing the other abnormal hand. The toe deformities were minor and would have been difficult to discern by antenatal ultrasound examination. The finding of a female fetus was a reassuring sign. To our knowledge, this is the first case report of finasteride use during pregnancy in a human. It is not clear if these deformities are related to finasteride use in pregnancy, but it is worthwhile to document a possible association and focus attention on the possibility of limb deformities in such cases.
